# Use of a real-time location system to detect cows in distinct functional areas within a barn

**DOI:** 10.3168/jdsc.2020-0050

**Published:** 2021-06-23

**Authors:** J.M. Chapa, L. Lidauer, A. Steininger, M. Öhlschuster, T. Potrusil, M. Sigler, W. Auer, M. Azizzadeh, M. Drillich, M. Iwersen

**Affiliations:** 1 FFoQSI GmbH—Austrian Competence Centre for Feed and Food Quality, Safety and Innovation, Technopark 1C, 3430 Tulln, Austria; 2 Clinical Unit for Herd Health Management in Ruminants, University Clinic for Ruminants, Department for Farm Animals and Veterinary Public Health, University of Veterinary Medicine Vienna, 1210 Vienna, Austria; 3 Smartbow GmbH, 4675 Weibern, Austria; 4 Department of Clinical Sciences, Faculty of Veterinary Medicine, Ferdowsi University of Mashhad, Mashhad, 9177948974, Iran

## Abstract

Automated sensor-based monitoring of cows has become an important tool in herd management to improve or maintain animal health and welfare. Location systems offer the ability to locate animals within the barn for, for example, artificial insemination. Furthermore, they have the potential to measure the time cows spend in important areas of the barn, which might indicate need for improvement in the management of the herd or individuals. In this study, we tested the sensor-based real-time location system (RTLS) Smartbow (SB, Smartbow GmbH) under field conditions. The objectives of this study were (1) to determine the accuracy of the system to predict the location of the cow and the agreement between visual observations and RTLS observations for the total time spent by cows in relevant areas of the barn and (2) to compare the performance of 2 different algorithms (Alg1 and Alg2) for cow location. The study was conducted on a commercial Austrian dairy farm. In total, 35 lactating cows were video recorded for 3 consecutive days. From these recordings, approximately 1 h was selected randomly each day for every cow (3 d × 35 cows). Simultaneously, location data were collected and classified by the RTLS system as dedicated to the alley, feed bunk, or cubicle on a 1-min resolution. A total of 6,030 paired observations were derived from visual observations (VO) and the RTLS and used for the final data analysis. Substantial agreement of categorical data between VO and SB was obtained by Cohen's kappa for both algorithms (Alg1 = 0.76 and Alg2 = 0.78). Similar results were achieved by both algorithms throughout the study, with a slight improvement for Alg2. The ability of the system to locate the cows in the predefined areas was assessed, and the results from Alg2 showed sensitivity, specificity, and positive predictive value of alley (74.0, 91.2, and 76.9%), feed bunk (93.5, 86.2, and 89.1%), and cubicle (90.5, 83.3, and 95.4%) and an overall accuracy of 87.6%.The correlation coefficient (r) between VO and SB for the total time cows spent (within 1 h) in the predefined areas was good to strong (r = 0.82, 0.98, and 0.92 for alley, feed bunk, and cubicle, respectively). These results show the potential of the system to automatically assess total time spent by cows in important areas of the barn for indoor settings. Future studies should focus on evaluating 24-h periods to assess time budgets and to combine technologies such as accelerometers and location systems to improve the performance of behavior prediction in dairy cows.

## Highlights

• The RTLS achieved high accuracy in locating cows in alleys, feed bunk and cubicles.
• Location and time spent in important barn areas can be automatically determined and used as indicators of health.
• The potential of combining RTLS with other sensors technologies was discussed.

## Graphical Abstract

**Figure:**
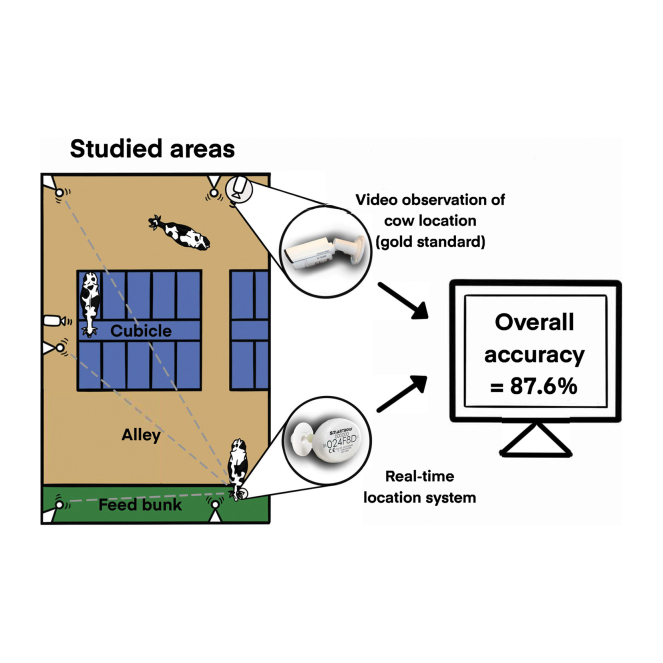
**Summary:** Cow location and time spent in relevant areas of the barn are important parameters that can be indicative of health and welfare status. Real-time location systems (RTLS) have the potential to measure these parameters on an individual level. We evaluated the ability of the Smartbow (Smartbow GmbH) RTLS to locate and measure the time cows spent in relevant areas of the barn. This work demonstrated that RTLS are capable of identifying individual cows in important areas of the barn and consequently are able to measure time budgets of cows.

Interest in the development of sensor technologies that allow monitoring of physiological (e.g., pH of rumen fluid, percentage of milk fat) and behavioral (e.g., rumination time, activity level) parameters continues to increase. This trend can be attributed in part to increases in herd size (Barkema et al., 2015) and the facilitation of herd and health management (Rutten et al., 2013) by monitoring individuals within such herds. Precision dairy farming systems include automated milking systems and automated feeders as well as sensors (e.g., pedometers, accelerometers) that can be mounted on the cows' legs, collars, or ears or placed in the rumen. Additionally, precision dairy farming may include real-time location systems (**RTLS**) that allow tracking of animals within a barn. These systems can be useful for detecting cows within the barn for, for example, AI as well as for estimating and predicting the time animals spend in relevant areas, such as the alley, feed bunk, or cubicle. Location data can also be used to predict the activity performed by animals in important areas of the barn (Shane et al., 2016) or specific behaviors, such as less time lying down and more time walking during estrus (Zebari et al., 2018) or longer lying time when moderately lame (Weigele et al., 2018).

Location systems can use several technologies, such as ultra-wide band (Porto et al., 2014), wireless sensor network (Huhtala et al., 2007), Bluetooth (Tøgersen et al., 2010), ultra-high frequency (Ipema et al., 2013), or global positioning systems (Guo et al., 2009). Computer vision also has been used for tracking and measuring activity in cows (Porto et al., 2013) and pigs (Ahrendt et al., 2011). Computer vision systems have the ability to assess other aspects of animal health and welfare, such as lameness (Viazzi et al., 2014) or BCS (Azzaro et al., 2011; Bercovich et al., 2013). In our study, we used the Smartbow system (**SB**; Smartbow GmbH), which consists of an accelerometer (3-dimensional) and location (2-dimensional) sensor fixed in an ear tag. Previous studies have evaluated the capabilities of the system to detect rumination (Reiter et al., 2018), predict calving (Krieger et al., 2017), detect estrus (Schweinzer et al., 2019), and monitor drinking events in calves (Roland et al., 2018). Additional studies have evaluated the location feature by focusing on the system's distance (in meters) accuracy in a cow barn (Wolfger et al., 2017), for dairy cows on pasture (Byrne et al., 2019), and for pigs maintained in a gestation stall (Will et al., 2017). However, the system's accuracy in predicting cow location and observing the total time cows spend in relevant areas of the barn (alley, feed bunk, and cubicle) has not been evaluated.

Time spent in relevant areas of the barn and cow location are important parameters that can be indicative of health and welfare status (Gomez and Cook, 2010). Real-time location systems can measure these parameters automatically and provide data for early detection of behavior changes relevant to a cow's health and welfare. Furthermore, sensors that combine technologies (accelerometer and location) are promising tools because they have the potential to collect multiple parameters, thus improving algorithm performance to classify behaviors (Wang et al., 2018). The objectives of this study were (1) to determine the accuracy of the system in predicting cow location and the agreement between visual observations (**VO**) and observations of the RTLS for the total time spent by cows in relevant areas of the barn and (2) to compare the performance of 2 algorithms (**Alg1** and **Alg2**) for cow location.

This study was conducted on a commercial Austrian dairy farm in May 2019. The farmer gave his informed consent to the use of the accelerometer data and video recordings of his animals. In total, 40 lactating cows (39 Brown Swiss and 1 Holstein Friesian) were housed in a freestall barn. Cows were milked twice a day (0800 and 1700 h), and fresh feed was offered once a day as a TMR at approximately 0830 h and pushed up 2 to 3 times during the day. The barn had 41 cubicles (dimensions: 2 m × 1.2 m; 19 bedded with sawdust and 22 with straw), a 30 m × 1 m feed bunk with 43 headlocks (~95% stocking density during the data collection), 2 concentrate feeders (dimensions: 2.8 m × 1.0 m), and 3 water troughs (dimensions: 1.1 m × 0.5 m). The alleys were 2.6 m wide between cubicles and 4.3 m wide between cubicles and the feed bunk (Figure 1).

**Figure 1:**
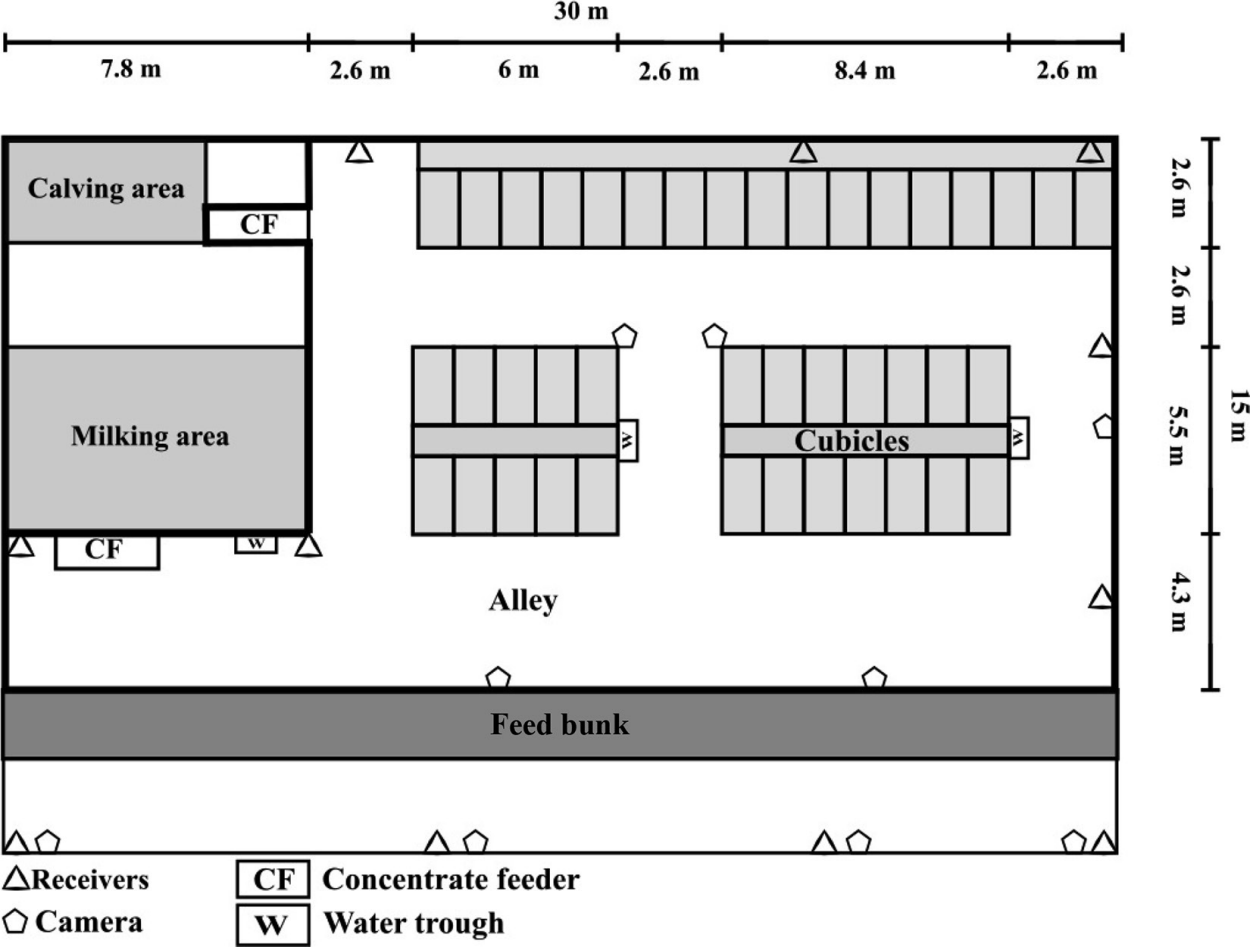
Floor map of the freestall barn used in the experiment.

In this study, the real-time location function of the SB system was used. The system is able to generate 2-dimensional location data and 3-dimensional acceleration data. Acceleration data were not used in this study. The system consists of ear tags (52 × 36 × 17 mm; weight = 34 g) that were attached to the cows' left ear and 10 receivers (Smartbow Wallpoints) that were installed around the barn (approximately 9 m apart and at a height of 3.4–2.2 m). The receivers collected data from the ear tags and sent them to the local system server (Smartbow Farm Server), where the data were processed. For the purpose of this study, a digital map of the barn in the system software was used and divided into 3 customized areas (alley, feed bunk, and cubicle; Figure 1). Concentrate feeders and water troughs were not included due to their small size (accuracy distance reported: mean distance difference of 1.22 m; Wolfger et al., 2017). The system algorithm calculated the time difference of arrival—that is, the time it took the signal to travel from the ear tags to at least 4 receivers and triangulate the location of the cow within the barn. Further information on the localization feature of the SB system is found in Byrne et al. (2019) and Sturm et al. (2020).

In this study, in addition to the algorithm already implemented in the SB software (Alg1), a more advanced algorithm (Alg2) developed by the SB manufacturer was tested for future use. In Alg1, a 2-dimensional Kalman filter is used to produce a filtered position from the current position raw data, taking into account the previous position of the animal. As an extension to Alg1, Alg2 also takes into account the current acceleration values of the SB ear tag and information on the farm-specific barn layout, which is stored as a map in the software. It can be assumed that this additional information can improve the accuracy of position determination because, for example, a cow with currently registered acceleration data is more likely to be standing or walking in an alley and not be lying in a cubicle. In the medium term, the manufacturer plans to integrate Alg2 into the SB software. The processed information was then categorized automatically by the system into one of the 3 predefined areas of the digital map. For the purpose of this study, 1 SB observation was generated every minute by the system as hh:mm per area for each animal in a Microsoft Excel file (Microsoft Corp.).

For VO, cows were marked with numeric symbols on both flanks for identification on video recordings. In total, 9 cameras were installed (IR Bullet Network Camera Version DS-2CD2642FWD-IZS, Hikvision) to observe all angles of the barn. Video recordings of approximately 1-h segments from 0900 to 1600 h (to avoid milking times, as the milking area lacked receivers) were collected over 3 d (May 28–30, 2019) and stored (21 h). Of these records, 1 h per animal was selected randomly for each day (35 cows × 1 h × 3 d) for a total of 105 h (i.e., 6,300 min) of video recordings. For labeling of the videos (gold standard), Mangold Interact software (version 18.0.2, Mangold International GmbH) was used to visually categorize the cow's position to one of the predefined locations (alley, feed bunk, or cubicle) at the beginning of every minute (hh:mm/area). Classification was done by one labeler. Classifications of cow location were alley (cow's head and 4 legs are in the alley), feed bunk (cow's head is completely behind the head lock), and cubicle (cow's head and at least the front 2 legs are inside the cubicle). Video recordings were synchronized with the RTLS system. Additionally, the RTLS system created “unknown” data when the system was unable to assign the location of the cow and “no data” when the system did not receive data from the ear tag. These observations were included in the final data analysis. The final data output was presented in Excel files as hh:mm per area for VO and SB and for each animal. Furthermore, we collected 2 sets of SB data outputs from 2 different algorithms (Alg1 and Alg2) to compare their performance.

From the 105 h of VO and SB available, 4.5 h (274 and 270 min for Alg1 and Alg2, respectively) was excluded for reasons such as “video length was 58 or 59 min instead of 1 h” (10 min), “cow lying between alley and cubicle” (126 min), “cow went to the milking parlor between the observation periods” (43 min), and the difference between algorithms for “cow inside the concentrate feeder” (95 min for Alg1 and 91 min for Alg2). For the final data analysis, 100.5 h (6,026 and 6,030 min for Alg1 and Alg2, respectively) of VO and SB was eligible.

Statistical analyses were performed using SPSS (version 26, IBM Corp.). Minute-by-minute agreement between both algorithms as well as the agreement between Alg1 or Alg2 and VO were evaluated by calculation of Cohen's kappa (κ; Cohen, 1960). The McNemar–Bowker test was used to detect significant differences between Alg1 and Alg2, which was defined as *P* < 0.05. Furthermore, a confusion matrix approach was used to visualize the classification of VO and SB (Alg1 and Alg2) as true positives, true negatives, false positives, or false negatives. Consequently, sensitivity, specificity, positive predictive value, and accuracy of the RTLS were calculated for each area of interest (i.e., alley, feed bunk, and cubicle). Total time spent at every predefined area was obtained from the sum of the minutes observed during each observation period for VO and SB (e.g., 20 min for alley, 20 min for feed bunk, and 20 min for cubicle). Normal distribution of total time (min/h) spent by cows at every area of interest obtained by the 2 methods was checked using the Shapiro–Wilk test. To evaluate the agreement of the total time spent (min/h) in each area during every SB and VO, nonparametric Spearman correlation coefficients were calculated. Bland–Altman (Bland and Altman, 1986) plots were used to evaluate the agreement between the 2 methods and to show the pattern of difference in total time spent (min/h) by cows in each area by the 2 methods. Furthermore, the statistical software R (R Development Core Team; version 4.0.3, packages chillR_0.72.2 and DescTools_0.99.39) was used to calculate the concordance correlation coefficient and the root mean square error of the total time spent in each area.

Two cows were excluded due to ear tag malfunction, and 3 were excluded because painted numbers were not visible at the time of video labeling. Cows used in the final data analysis (8 primiparous and 27 multiparous) were at lactation 3.2 (range: 1–8) and 289 ± 134 DIM (mean ± SD). Average time of observation per hour was 57.4 min due to the excluded minutes, as previously explained.

Similar results were found for both algorithms. For this reason, only results for Alg2 are explained in the text, and results for both algorithms are presented in Table 1, Table 2. From the 6,030 VO (taken from 0900 to 1600 h), 21, 19, and 59% were in the alley, feed bunk, and cubicle, respectively, whereas 20, 20, and 56% of SB observations were associated with these areas, respectively (Table 2); the remaining 4% corresponded to “unknown data” and “no data” observations. On the SB, we observed an increased number of false positives and false negatives in the alley (23 and 25%, respectively) compared with the feed bunk (10 and 6%, respectively) and cubicle (4 and 9%, respectively; Table 2). This may be due to the distance between the studied areas; the alley is between the feed bunk and cubicles, and thus the likelihood of predicting false positives and false negatives for the alley is increased. Furthermore, we observed that cows closer to the borders of areas (corner cubicles) had an increased number of false positives and false negatives. Concentrate feeders were initially included in the data analysis; however, due to the small number of observations (VO 1.5% and SB 0.06%) and the small area of the feeders, the system obtained poor results (sensitivity = 0.03, specificity = 0.87, positive predictive value = 0.75, and accuracy = 0.86 for Alg2). Hence, we excluded this area from the final analyses.

**Table 1:** Overall results for cows' total time spent in different locations in the barn by real-time location system (RTLS) classification from algorithm 1 (Alg1) and algorithm 2 (Alg2) compared with visual observation^1^

Location	Algorithm	Sensitivity, %	Specificity, %	RMSE	CCC	r	Bland–Altman		
							MD	SD	95% CI
Alley	Alg1	71.7	90.1	7.75	0.80	0.79**	−0.40	7.78	−15.66 to 14.85
	Alg2	74.0	91.2	6.10	0.87	0.82**	−0.21	6.12	−12.22 to 11.79
Feed bunk	Alg1	93.1	86.2	2.31	0.98	0.98**	−0.43	2.28	−4.90 to 4.03
	Alg2	93.5	84.5	2.55	0.98	0.98**	−0.64	2.48	−5.52 to 4.23
Cubicle	Alg1	90.5	83.3	9.09	0.91	0.90**	1.65	8.98	−15.96 to 19.27
	Alg2	89.1	81.9	7.63	0.93	0.92**	1.73	7.47	−12.91 to 16.37

1 RMSE = root mean square error; CCC = concordance correlation coefficient; r = Spearman correlation; MD = mean deviation.

** *P* ≤ 0.01.

**Table 2:** Results for classification of visual observations (VO) and real-time location system (RTLS) observations for algorithm 1 (Alg1) and algorithm 2 (Alg2)^1^

RTLS	VO						Total	
	Alley		Feed bunk		Cubicle			
	Alg1	Alg2	Alg1	Alg2	Alg1	Alg2	Alg1	Alg2
Alley	**912**	**941**	74	52	256	230	1,242	1,223
Feed bunk	117	130	**1,084**	**1,089**	1	2	1,202	1,221
Cubicle	213	155	2	1	**3,201**	**3,254**	3,416	3,410
Unknown	29	45	3	21	105	76	137	142
No data	0	0	1	1	28	33	29	34
Total	1,271	1,271	1,164	1,164	3,591	3,595	6,026	6,030

1 Bold values represent true-positive classifications.

Categorical classifications of cow location (alley, feed bunk, and cubicle) were recorded on a minute basis by VO and compared with the corresponding minute recorded by SB. The results of Cohen's kappa test showed substantial agreement (Landis and Koch, 1977) between VO and SB, with κ = 0.78 for Alg2. The agreement of observations between Alg1 and Alg2 was κ = 0.84. Both algorithms showed different classifications of cow location (*P* < 0.001). From the paired data of VO and SB (Alg1 and Alg2), a confusion matrix was performed; test characteristics for each area are presented in Table 1, Table 2. Results from Alg2 showed sensitivity, specificity, and positive predictive value of alley (74.0, 91.2, and 76.9%), feed bunk (93.5, 86.2, and 89.1%), and cubicle (90.5, 83.3, and 95.4%) and an overall accuracy of 87.6%. The total time spent (min/h) per cow in the specific areas during the VO and SB observed period was derived from the sum of the classification of the categorical data. Spearman correlation coefficients (r) for VO and SB regarding total time each cow spent in each area were 0.82, 0.98, and 0.92 for alley, feed bunk, and cubicle, respectively (Table 1). Bland–Altman analysis was used to determine agreement between VO and SB for total time spent in each area. To assess the agreement of the continuous data of VO and SB, the concordance correlation coefficient obtained results of 0.87, 0.98, and 0.93 for alley, feed bunk, and cubicle, respectively. The root mean square error was used to estimate the error of the system to predict the total time spent in each area (6.10, 2.55, and 7.63 for alley, feed bunk, and cubicle, respectively; Table 1).

In this study, we tested the ability of the SB system to locate the cows at selected functional barn areas in a commercial setting and measured the time cows spent in these areas. When comparing the classification of cows' locations at a specific time by VO with those automatically done by RTLS Alg1 and Alg2, we found substantial agreement and high overall accuracy of the system in identifying where the cows were located (alley, feed bunk, or cubicle). There was significant improvement in categorizing cows' location with Alg2. These results allowed us to calculate the total amount of time spent in each area, which in turn had strong agreement with SB data. The distance accuracy of the SB system was previously evaluated with cows by Wolfger et al. (2017). In that study, the authors used static (ear tag attached to a stick) and dynamic (ear tag attached to the cow) measures from the system. Dynamic measures obtained a mean distance difference of 1.22 m. Using the same system, Will et al. (2017) evaluated the distance accuracy in a sow gestation stall. They found a Euclidian distance of 2.7 m, which was improved to 2.0 m by using a filter for noise on the signal caused by metal structures (9% average in data losses). In our study, however, we tested the accuracy of the system to locate and estimate the time spent at relevant areas of the barn rather than evaluating the distance accuracy of the system. Other studies with a similar approach used a wireless sensor network (CowView system; GEA Farm Technologies) to evaluate the location and activity of cows (Tullo et al., 2016). They reported accuracies of locating the cow in the alley and in the cubicle of 93 and 97%, respectively. Porto et al. (2014) used an RTLS based on ultra-wide band technology and found a mean error of 0.5-m distance. More recently, Hindermann et al. (2020) also obtained an accuracy of 0.5 m under optimal conditions. Global positioning system technology was also used in a paddock to determine the areas (10 × 10 m) more intensively used by cows (water area and edges of the paddock; Guo et al., 2009). Thus, using an RTLS can provide information about the locations of cows and how much time they spend in these areas. However, the activities and duration of them are unknown (Shane et al., 2016). In a study on calves, the probability of estimating drinking (median 54%) and eating (median 88%) activities when located at specific areas was assessed (Shane et al., 2016). Location data have also been used for more practical approaches. For example, data obtained from an ultra-wide band system were used to create a software tool to visualize the instant velocity of cows over time. This in turn could represent the pattern of estrus in the software tool (Arcidiacono et al., 2018). Furthermore, in other studies, location data have typically been combined with acceleration data in an attempt to increase the performance in behavior classification. Wang et al. (2018) demonstrated that combining these 2 technologies improved the classification of feeding behavior by 20%. Furthermore, combining location with accelerometer data may improve the automated assessment of health and welfare (Chapa et al., 2020). In a study by Barker et al. (2018), positioning of the cow and activity from acceleration data were combined to classify feeding behavior with an overall accuracy of 83%. With data derived from the system, they found that lame cows had less mean total daily feeding time than nonlame cows.

Some limitations of this study included the fact that the observation periods consisted of 1 h/d per animal. Although the correlations of time spent per area were high, assessing time budgets with short observation periods was not possible. It was not possible to classify small areas such as concentrate feeders and water troughs with the system. Hence, additional technologies (e.g., antennas or radio frequency identification) are needed to detect animals in these functional areas.

In this study, the SB system was able to classify the location of animals in predefined areas (e.g., alley, feed bunk, and cubicle) with sufficient accuracy but failed to detect animals at the concentrate feeder. The total amount of time a cow spent at certain areas within the observed period was strongly correlated with the location data of the system. This could be adapted into 24-h periods to assess time budgets and thus be used for research or management purposes. Further studies should focus on using longer time periods to identify time budgets of cows using the location system and on algorithm development and validation of sensors that are able to collect different types of data (acceleration and location). Furthermore, combining technologies in a single sensor (accelerometer and location) may enable the systems to collect a wider range of behavioral parameters, which can improve the accuracy of predicting behaviors.
