# Erratum to “Use of a real-time location system to detect cows in distinct functional areas within a barn” (JDS Commun. 2:217–222)

**DOI:** 10.3168/jdsc.2022-3-2-163

**Published:** 2022-03-16

**Authors:** J.M. Chapa, L. Lidauer, A. Steininger, M. Öhlschuster, T. Potrusil, M. Sigler, W. Auer, M. Azizzadeh, M. Drillich, M. Iwersen

The animal ethics statement on page 218 should be revised as follows (addition in bold): “This study was conducted on a commercial Austrian dairy farm in May 2019. The farmer gave his informed consent to the use of the accelerometer data and video recordings of his animals. **For the study, the animals were observed exclusively by video cameras and no manipulations of the animals were necessary. Therefore, ethical approval for animal experimentation by an institutional animal care and use committee was not considered necessary in our jurisdiction.**”
